# Supplementary material for: Flexible linkers in CaMKII control the balance between activating and inhibitory autophosphorylation
Source: eLife. 2020 Mar 9;9:e53670. doi: 10.7554/eLife.53670 (PMC7141811; doi:10.7554/eLife.53670)
Supplement: Source code 1. — A readme file and a test dataset is included for clarity. [file elife-53670-code1.zip › source-code-file/README.docx]

**README for Matlab codes and analysis**

1. Get all the raw image files in .tif format in a folder of your choice. Generate a pairwise composite image (488:560 channels or 488:640 channels) using virtual stack in ImageJ. A composite file is provided as a test dataset.

2. Use the code CAMKII_ImageJ_Pretreatment_Duplication_v2_TwoChannel_WOBlank.m to reconstruct duplicate images for tracking in the TrackMate module of ImageJ (check code comments for details). These should write out files Tirf488.tif (for 488 channel) and Tirf560.tif (for 560 channel).

**Note**: If you have a 488:640 composite pair, the output files are still called Tirf488.tif and Tirf560.tif, where Tirf560.tif represents data for the 640 channel.

3. Single particles were detected by analyzing these files (Tirf488.tif and Tirf560.tif) using the single particle tracking plugin TrackMate in ImageJ (Jaqaman et al., 2008). These will generate spot and track statistics for each single particle, which are written out as .csv files.

4. Append the channel name to the names of these .csv files (e.g., Spots in tracks statistics488.csv and Track statistics488.csv).

4. These spot and track statistics .csv files are inputs to the code find_coloc_plot_intensity.m, which calculates the fraction of colocalization and plots the intensity histograms (check code comments for details).

A **test dataset** is provided in the sub-folder called **composite.tif**.
